# Antibody response dynamics to the *Plasmodium falciparum *conserved vaccine candidate antigen, merozoite surface protein-1 C-terminal 19kD (MSP1-19kD), in Peruvians exposed to hypoendemic malaria transmission

**DOI:** 10.1186/1475-2875-7-173

**Published:** 2008-09-09

**Authors:** Katherine J Torres, Eva H Clark, Jean N Hernandez, Katherine E Soto-Cornejo, Dionicia Gamboa, OraLee H Branch

**Affiliations:** 1 Instituto de Medicina Tropical "Alexander von Humboldt," Universidad Peruana Cayetano Heredia, Lima, Peru; 2 Department of Medicine, Division of Infectious Disease, University of Alabama, Alabama, Birmingham, USA

## Abstract

**Background:**

In high-transmission areas, developing immunity to symptomatic *Plasmodium falciparum *infections requires 2–10 years of uninterrupted exposure. Delayed malaria-immunity has been attributed to difficult-to-develop and then short-lived antibody responses.

**Methods:**

In a study area with <0.5 *P. falciparum *infections/person/year, antibody responses to the MSP1-19kD antigen were evaluated and associations with *P. falciparum *infections in children and adults. In months surrounding and during the malaria seasons of 2003–2004, 1,772 participants received ≥6 active visits in one study-year. Community-wide surveys were conducted at the beginning and end of each malaria season, and weekly active visits were completed for randomly-selected individuals each month. There were 79 *P. falciparum *infections with serum samples collected during and approximately one month before and after infection. Anti-MSP1-19kD IgG levels were measured by ELISA.

**Results:**

The infection prevalence during February-July was similar in children (0.02–0.12 infections/person/month) and adults (0.03–0.14 infections/person/month) and was negligible in the four-month dry season. In children and adults, the seroprevalence was maintained in the beginning (children = 28.9%, adults = 61.8%) versus ending malaria-season community survey (children = 26.7%, adults = 64.6%). Despite the four-month non-transmission season, the IgG levels in *Plasmodium*-negative adults were similar to *P. falciparum*-positive adults. Although children frequently responded upon infection, the transition from a negative/low level before infection to a high level during/after infection was slower in children. Adults and children IgG-positive before infection had reduced symptoms and parasite density.

**Conclusion:**

Individuals in low transmission areas can rapidly develop and maintain αMSP1-19kD IgG responses for >4 months, unlike responses reported in high transmission study areas. A greater immune capacity might contribute to the frequent asymptomatic *P. falciparum *infections in this Peruvian population.

## Background

The prospect of decreasing millions of *Plasmodium falciparum *malaria deaths each year [1,2] hinges upon the development, implementation and evaluation of vaccines. A successful strategy requires an appreciation of the human-host immune response development upon natural infection. Longitudinal studies in hyperendemic malaria transmission areas have elucidated factors related to the development – or lack of development – of antibody responses [3-10]. Due to overlapping malaria infections in high transmission areas, where infections occur monthly or even daily, it is difficult to study the immunological responses elicited upon and maintained after a discrete infection. Additionally, studies in high transmission areas have a limited ability to consider age because participants suffer many infections during their first five years of life [11].

For such reasons, the Peruvian Amazon, a recent and low malaria transmission region, offers unique research opportunities. *P. falciparum *emerged in this area in 1994, and fewer than 0.42 infections/person/year (with transmission occurring seven months out of the year) have been detected since 2000, even when using active case detection (ACD) in the most at-risk age groups (12–30 years old) living in the highest transmission zones [12]. Despite this low and recent transmission, there is an abundance of non-febrile infections [12,13]. A 2005 study of 72 infections demonstrated that 45 of these infections remained asymptomatic, with no indication of fever, anaemia, or parasitaemia greater than 5,000 parasites/μl of blood for one week[12]. Surprisingly, 15 of these 45 asymptomatic individuals were *P. falciparum*-negative one week later, were not treated, and remained without microscopy-detectable *P. falciparum *parasites for at least one month [12].

In the current study, the antibody responses to the 19kD fragment of *P. falciparum *Merozoite Surface Protein-1 (MSP1-19kD) are characterized. MSP1-19kD is one of the most well studied malaria vaccine candidates. Experimental infection studies and *in vitro *and animal model studies have demonstrated an association between αMSP1-19kD antibodies and protection from clinical infection [5,14-18]. The protective effects of antibodies to MSP1-19kD are postulated to occur by inhibiting the proteolytic cleavage of MSP-1 needed to expose the 19kD fragment and/or by blocking the MSP1-19kD from binding to erythrocytes. Immuno-epidemiologic studies suggest that αMSP1-19kD IgG responses are associated with reduced clinical symptoms, reduced parasitemia, and reduced risk of subsequent infection [4,5,7,9,19]. However, the fact that immunity does not develop until years after exposure to this highly conserved antigen brings the vaccine candidacy of MSP1-19kD into question. In fact, although possibly explained by a myriad of factors, a recent clinical vaccine trial using a 42kD fragment of MSP1 (19kD along with MSP1's upstream 33kD fragment) was not significantly associated with protection.

This investigation was prompted by prior studies in high transmission regions that suggested a slow or difficult-to-develop αMSP1-19kD response (requiring high parasitaemia and/or frequent infection) which, when developed, is short-lived, lasting less than six months[3,6,7,10], even in individuals >5 years old[3,4,6,7,10,21,22]. Therefore it was predicted that αMSP1-19kD responses in Peruvian individuals would be infrequent or absent before each widely-spaced infection. On the contrary, this study suggests that this population has a high capacity for eliciting and maintaining αMSP1-19kD IgG responses. This immune capacity may contribute to the high frequency of asymptomatic *P. falciparum *infections observed in this Peruvian population [12,13].

## Methods

### Study area and participants

The Malaria Immunology and Genetics in the Amazon (MIGIA) study takes place in a community called Zungarococha (there are 1907 individuals enrolled in our study from this community) with a maximum *P. falciparum *infection rate of 0.42 infections/person/year (details in Branch *et al *[12]).

In 2003 and 2004 active case detection (ACD) was undertaken during the malaria transmission season (February – July) and passive case detection (PCD) was performed throughout the year. ACD was conducted in two different ways. In three community-wide surveys as many community members as possible were sampled using home-based visits: March 2003 (N = 363), February 2004 (N = 1156), and August 2004 (N = 861). Additionally, each month during malaria season (April-July 2003 and also March-July 2004), approximately 220 community-members were randomly selected from 100-meter regions surrounding homes where *P. falciparum *was detected during the prior month and were enrolled for one month of weekly home-based visits. There were 1,722 participants who completed at least four ACD visits in one malaria season: 671 in 2003 and 1051 in 2004, of which 423 completed at least four ACD visits both in 2003 and 2004.

Additionally, throughout 2003 and 2004 infections were detected via PCD at the local health post. All individuals entering the Zungarococha health post complaining of febrile illness or other malaria-like symptoms had a blood slide read by an expert microscopist. Malaria treatment is free and tightly controlled (observed therapy), and there is no access to private physicians.

At each ACD and PCD, a detailed clinical and epidemiologic evaluation was made by a trained physician and blood was collected (0.25 ml in ACD and 3–6 ml in PCD). Thin and thick blood-smears were made, haematocrit capillary tubes were filled, and blood was separated into packed erythrocytes and sera and stored at -70°C.

### Human subjects approval

All individuals in the community were invited to participate in the ACD study protocol and clinic protocol if they were diagnosed with malaria either in ACD or PCD. Ethical clearance was received from the University of Alabama at Birmingham, the Universidad Peruana Cayetano Heredia (Lima, Peru), and the Ministry of Health (MINSA, Lima, Peru).

### Diagnosis

Thick blood smears were examined using Giemsa staining and oil-immersion microscopy by three highly qualified microscopists. If >50 ring-stage parasites were detected after reading 200 leukocytes, parasitaemia was calculated from these fields. Otherwise, at least 500 leukocytes were counted. Parasitaemia was expressed as the number of parasites/μL of blood, assuming 6,000 leukocytes/μL. The blood-smear was considered negative if no parasites were seen in 200 fields.

### Treatment

Whether in active or passive case detection, all treatments were given through the MINSA authorities, following the MINSA National Drug Policy Guidelines. *Plasmodium vivax *treatment is chloroquine (10 mg/kg for three days) with primaquine (0.5 mg/kg for seven days). *Plasmodium falciparum *treatment is mefloquine (12.5 mg/kg daily for two days) with artesunate (4 mg/kg daily for three days) in non-pregnant patients older than one year of age.

### Measurement of IgG

The recombinant *P. falciparum *MSP1-19kD, corresponding to Ugandan-PA *P. falciparum *strain (with the MSP1-19kD 4 aa variant positions of E-K-N-G)[23], was expressed in *Sacharomyces cerevisiae *(provided by Dr. David Kaslow). The variants E-K-N-G and Q-K-N-G are the only *P. falciparum *MSP1-19kD detected in this population. One hundred and twenty eight randomly selected samples were tested (by ELISA). The anti-EKNG and anti-QKNG IgG responses (Negative/Low Positive/High Positive) were nearly identical (concordance = 92%).

An enzyme-linked-immunoabsorbent assay (ELISA) for total IgG was performed as described previously [7]. Briefly, plates (Dynatech) were coated with 50 μL/well of recombinant MSP1-19kD (at a concentration of 0.5 ng/μL) and blocked with 1% bovine serum albumin (BSA). The sera were diluted 1:100 (1.5% non-fat milk in washing-solution [0.15 M Na_2_HPO_4_, 0.15 M NaH_2_PO_4_, NaCl, 0.05% Tween20, and 0.05% BSA]). The IgG that bound to the MSP1-19kD on the plate were detected with peroxidase-conjugated goat-antihuman-IgG (Chemicon) diluted 1:2000. The plates were washed, 50 μL/well of 3,3',5,5'-tetramethylbenzidine (KPL) was added, the reaction was stopped using 50 μL/well of 0.25 M HCL, and then read at absorbance 450 nm (A_450_) with an ELISA plate-reader (BIORAD).

Ten serum samples from healthy non-exposed Peruvians were used as negative controls. The positive controls included five samples from five different *P.falciparum*-infected individuals and a "positive pool" made-up of three samples.

A positive pool control standard-curve was made for each experiment day. The positive pool was diluted at 1:50, 1:100, 1:400, 1:1600, 1:3200, and 1:12800. The results from each day showed similar curves. The average positive pool optical density (OD) at a 1:400 dilution for *all *experiment days was subtracted from the positive pool OD for each individual experiment day. The resulting correction factor was subtracted from the initial OD of each sample, thereby standardizing by experiment day.

Samples with an OD greater than the negative cutoff (the average of the negative controls plus 2 standard deviations) were considered positive. The positive group was divided into "Low" and "High," where an OD greater than 3 times the negative cutoff was called "High-Positive."

### Study design and statistical analysis

Of 1,722 individuals, 600 individuals were selected who participated in at least one ACD community survey: 300 children (1–14.5 years-old) and 300 adults (14.6–104 years-old). Of these 600 age-grouped and then randomly selected individuals, 448 participated in 2 of the 3 community surveys, and 49 participated in all three community surveys. There were a total of 868 sample-events in the three community surveys.

Seventy-nine *P. falciparum *infections (from 79 different individuals) were selected from the 182 infections detected in ACD because these represented individuals who had sera samples available from before, during, and after their infection. Forty-one of these were adult infection samples.

"Before", "During", and "After" infection time points were selected. From samples collected within 3 months before infection, the sample closest to 1 month before was selected as "Before": median days until infection (1^st^–3^rd ^quartile) = 38 days (14–68). From samples collected within 3 months after infection, the sample closest to 1 month after was selected as "After": 22 days (14–53). The before, during, and after sera samples were encountered for 79 individual infections.

An infection was classified as "symptomatic" if the individual had a fever greater than or equal to 38.3°C, reported a fever in the past 2 days, or had a haematocrit of less than 30 PCV either at the time of or within one week after infection.

Statistical analysis was conducted using SAS (SAS Cary, NC, version 9). Chi square (χ^2^) analysis tested infection and seroprevalence differences. A non-parametric Wilcoxon Rank Sums (WRS) tested antibody level differences. A general linear model (GLM with General Estimating Equations (GEE) obtained by SAS procedure "GENMOD") determined variables associated with antibody level changes upon infection. The independent variables considered were age, logarithmic parasite density, sex and days separating the measurements of IgG. The GLM dependent variable was the IgG OD "During" minus the IgG OD "Before" time point. When testing frequency of fever with antibody response description over time (response profile), a Fisher's Exact and Mantel-Haenszel Chi-Square was used. Logistic regression analysis (also using GENMOD) determined if the above-listed variables could predict symptomatic infections, using risk estimates calculated by the GEE.

## Results

### *Plasmodium falciparum *prevalence and αMSP1-19kD IgG seroprevalence in three community surveys

**Figure 1:**
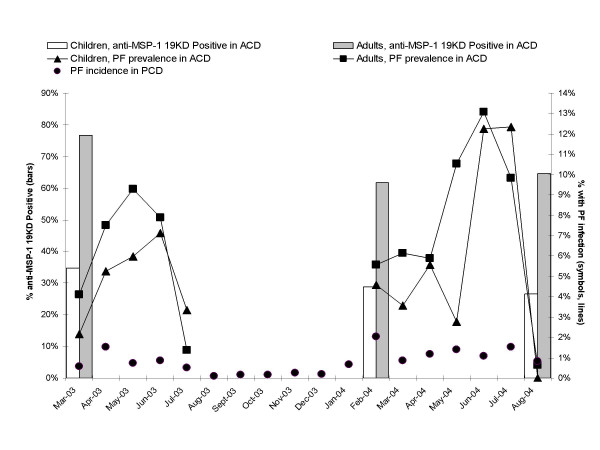
***P. falciparum *incidence and αMSP1-19kD prevalence in our study area between 2003–2004**. αMSP1-19kD IgG seroprevalence during March 2003, and February and August 2004, active case detection community surveys (bars) as well as *P. falciparum *point-prevalence during community surveys (open symbols) and weekly ACD conducted on sentinel individuals April-July, 2003, and March-July, 2004 (closed symbols, lines), and *P. falciparum *incidence in passive case detection (PCD) are shown (closed circles).

Infection prevalence (Figure 1) was calculated during the ACD community surveys (point prevalence) and also during the month of weekly ACD (monthly prevalence) for each individual. Each month, adults usually demonstrated a higher frequency of infection than children (Figure 1); however, this was not significantly different (χ^2^, p = 0.0901). The study-period PCD data as well as the historic records and the entomologic data [12,24] agree that malaria infections rarely occur within the dry season of August through December. In addition, during a recent small community survey that was conducted specifically to verify that there is negligible transmission during the dry season, 238 blood samples were collected in November through December, 2007. This survey included 123 febrile individuals as well as 115 non-febrile family members who simply accompanied the febrile individual to the community health center (sampling these individuals fell within the same ethically-reviewed protocol as described in this manuscript). Of these 238 individuals, all were microscopy negative for malaria and only one was PCR (sub-microscopy) positive for *P. vivax*. None were PCR positive for *P. falciparum*.

Returning to our 2003–2004 data, as expected due to the dry season beginning in July, the infection prevalence was near zero in the August 2004 community survey (Figure 1). Even though there was a similar infection prevalence in adults and children, significantly fewer children were IgG positive, regardless of the community survey epoch, versus adults (χ^2^, p < 0.05 for each community survey, Figure 1). For example, in the 2003 malaria season community survey (March 2003), 34.8% of children and 76.8% of adults were IgG-positive (point seroprevalence).

Despite low transmission, the αMSP1-19kD seroprevalence in the beginning versus the end of the transmission season was similar (Figure 1). Although it is possible that some individuals had a malaria infection on or before the February 2004 survey, only a small proportion of the αMSP1-19kD positivity could be attributable to such a rare early-season malaria infection given the low transmission. Specifically, given the maximum *P. falciparum *transmission rate found in ACD and PCD, together, in the highest transmission zones of this community [12], the probability of infection during the 4 months between these two community surveys was only 0.06.

### αMSP1-19kD IgG responses are maintained

In both *Plasmodium*-negative adults and children, the frequency of having a High Positive IgG response varied by <5% between the community survey conducted at the beginning of the 2004 malaria season (February 2004) and that conducted at the end of the 2004 malaria season (August 2004) (χ^2^, p < 0.05; Table 1).

**Table 1:** αMSP1-19kD by age group. In *Plasmodium*-negative^a ^individuals, αMSP1-19kD IgG responses^b ^differed by age group^c^, but were similar within age group at the beginning versus end of the malaria season^d^.

**Malaria status**	**Age group**	**Anti-MSP-1 19KD IgG response stratification**	**Mar., 2003** **(%, n)**		**Feb., 2004** **(%, n)**		**Aug., 2004** **(%, n)**	
NEG	Adults	≤ negative cut-off	16.2%	33	38.2%	68	35.4%	40
NEG	Adults	> negative cut-off, but < 3 times negative cut-off	20.6%	17	13.5%	24	21.2%	24
NEG	Adults	≥ 3 times negative cut-off	63.2%	92	48.3%	86	43.4%	49
NEG	Children	≤ negative cut-off	54.2%	107	71.1%	118	73.3%	77
NEG	Children	> negative cut-off, < 3 times negative cut-off	23.2%	17	10.8%	18	10.5%	11
NEG	Children	≥ 3 times negative cut-off	22.6%	40	18.1%	30	16.2%	17
		p (chisq): age group by IgG response	< 0.0001		< 0.0001		< 0.0001	

^a^Malaria negative (NEG) = no *Plasmodium *infection (neither *P. falciparum *nor *P. vivax*) detected by microscopy or PCR.

^b^Antibody response stratification: Negative ≤ negative cut-off; High-Positive ≥ 3 times negative cut-off; Low-Positive > Negative and < High-Positive.

^c^Adults = ≥ 14 years old.

^d^February 2004 = beginning of transmission season. August 2004 = end of transmission season.

Several *Plasmodium*-negative individuals in the February 2004 survey were infected with *P. falciparum *at some time during the following malaria season. One-hundred and thirty-five individuals were evaluated who were *Plasmodium*-negative in February 2004 and also who were in both the February 2004 and the August 2004 community surveys (e.g., represented twice in Table 2). Seven of the 29 individuals who were IgG negative in February 2004 were infected between March and July, 2004. In August 2004, five of these recently (but not currently) infected individuals were αMSP1-19kD IgG positive. The only two individuals who were IgG negative, despite this recent infection, were children.

**Table 2:** Comparison of αMSP1-19kD IgG responses with malaria infection status during community surveys.

**Malaria status**	**Age group**	**N **(n < neg cut-off, n > 3* neg cut-off)	**Median**	**Lower quartile**	**Upper quartile**	**p **(WRS test)
PF	Adults	14 (1, 14)	1.446	1.141	1.851	0.3132
PF	Children	14 (3, 8)	1.339	0.740	1.803	
NEG	Adults	433 (141, 227)	1.040	0.505	1.562	<0.0001
NEG	Children	435 (302, 89)	0.301	0.161	0.827	

In adults, *P. falciparum *infected, *P. vivax *infected, and *Plasmodium*-negative (NEG) individuals had similar αMSP1-19kD IgG OD levels. The low αMSP1-19kD response frequency in NEG children suggests that fewer children had prior *P. falciparum *infection which could explain the maintained αMSP1-19kD antibody response seen in adults.

Similarly, *Plasmodium*-negative adults, but not children, had high median αMSP1-19kD levels (1.040 versus 0.301; Table 2). The *P. falciparum*-infected adult median αMSP1-19kD IgG level was not significantly different than that seen in *Plasmodium*-negative adults, and the majority of *Plasmodium*-negative adults had a high αMSP1-19kD IgG level (3^rd ^quartile = 0.505). The median IgG level in *P.falciparum*-infected children (1.339) was also similar to that seen in infected adults (Table 2). Although most *Plasmodium*-negative children had low αMSP1-19kD IgG levels, some had a high αMSP1-19kD IgG level (3^rd ^quartile = 0.827).

The αMSP1-19kD IgG level in *P. vivax*-infected individuals (detected in community surveys) was similar to that in *Plasmodium*-infected children (Table 2). The low homology between the *P. vivax *and *P. falciparum *MSP1-19kD [25] supports this observation.

### Age-related IgG dynamics before, during and after infection

**Figure 2:**
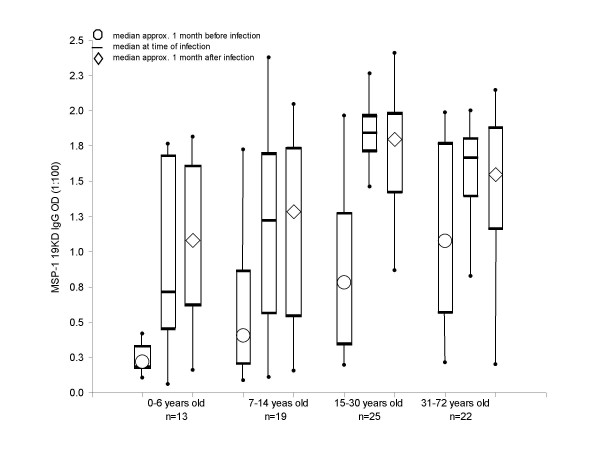
**αMSP1-19kD IgG level dynamics in 79 *P. falciparum *infections**. αMSP1-19kD IgG level dynamics in 79 *P. falciparum *infections: During, approximately one month Before and approximately one month After infection, are shown while grouping by age. The box-plot shows the median (symbols), first, and third quartile boundaries boxed off and data range (the whiskers are drawn not including the <3 outliers per group, although all data are included when calculating the median and quartiles).

Sera samples taken 'During' as well as approximately one month 'Before' and one month 'After' infection were evaluated to determine the dynamics of the αMSP1-19kD IgG antibody response and to see how this response changes between age groups (Figure 2). A positive αMSP1-19kD IgG response in the Before sample was observed in 36%, 69%, 84% and 85% of the 0–6, 7–14, 15–30, and >30 years-olds, respectively. In the 0–6 year-old group, the median αMSP1-19kD IgG was only 0.216 Before, but 0.712 During and then 1.08 After infection. Only the 0–6 year-old group had an increase in median αMSP1-19kD level after infection. The Before IgG level clearly increased with age. In the older age groups, there was little difference in median IgG level across During and After time points.

The antibody level change upon detection of an infection (which was predicted to be early in the infection due to our ACD) was evaluated by subtracting the Before from the During infection IgG OD in each individual (Figure 2). A step-down multivariate model of During-minus-Before showed that each age-group of individuals >6 years-old had a significantly greater αMSP1-19kD level change upon infection versus the 0–6 year-olds (GEE, p < 0.0418). None of the other variables tested (see methods) were associated with this detection-associated change in αMSP1-19kD level (p > 0.1).

### Response profile associated with fever and parasite density

The αMSP1-19kD response profile was defined for each individual infection by the responses measured in the Before, During, and After infection samples as follows:

#### Pre-Positive

An individual who was High Positive at all three time points or who was Low Positive Before infection and then became High Positive After infection.

#### Infection Positive

An individual who was IgG negative Before infection but then became High or Low Positive During and After infection.

#### Late Positive

An individual who was IgG negative Before and During infection, but then became High or Low Positive After the infection.

#### Non-Responder

An individual who did not have an antibody response at all or was only Low Positive during the infection and then became Negative after the infection.

Seventy-seven infections with complete clinical data could be classified via these groupings (Table 3).

**Table 3:** Adult and child IgG response profile as compared with clinical data during longitudinal follow-up.

**IgG response profile category**		**# with clinical information complete**	**% in each response category**	**# with febrile illness**	**% with febrile illness**	**GMP (95% CI)**	
**Adult**	Pre-Positive	22	48.9%	4	18.2%	19	(6–60)
	Infection-Positive	18	40.0%	9	50.0%	78	(37–164)
	Late-Positive	1	2.2%	0	0	16	n/a
	Non-responder	4	8.9%	3	75.0%	156	(12–2027)

**Child**	Pre-Positive	6	18.2%	1	16.7%	8	(1–53)
	Infection-Positive	16	48.5%	12	75.0%	284	(121–669)
	Late-Positive	4	12.1%	4	100.0%	1210	(754–1941)
	Non-responder	7	21.2%	6	85.7%	120	(26–561)

Adult and child IgG response profile was compared with febrile illness (patient-reported history or measured by physician within one week of infection) and parasite density, where infections were detected during one of 4 weekly active sampling visits. A pre-positive response, most frequently observed in adults, was associated with less febrile illness (χ^2^, p = 0.0056) and lower parasite density (WRS, p = 0.0005).

As shown in Table 3, the majority of adults were Pre-Positive (48.9%), while the majority of children were Infection Positive (48.5%). Fever was more frequent in children (58.1%) versus adults (26.1%) (Fisher's Exact Test, p = 0.0056). The geometric mean parasite density (GMP [95% confidence interval]) was higher in children than in adults (133 [218–509] versus 114 [71–185], WRS p = 0.0050). Within each age group, the response profile was associated with febrile illness (Mantel-Haenszel χ^2^, p = 0.0048 in adults and p = 0.0212 in children), where Pre-Positive had the lowest febrile frequency. Because the number of adults in each IgG response profile category was clearly different than children, multivariable logistic regression analysis was performed to investigate risk of febrile infection (presented below).

While including age, response profile, and parasite density, age group was no longer predictive of risk to symptomatic infection (GEE, p = 0.2415) and was removed from the model. Comparing to the Non-Responder profile, Pre-Positive, Infection Positive, and Late Positive each had an estimated risk of -2.23 (SE = 0.94), -0.61 (SE = 0.95), and 0.61 (SE = 1.3), respectively. There was a significant decrease in risk to symptomatic infection in Pre-Positive individuals versus Non-Responders (GEE, p = 0.0174). Independently, with each increase in log parasite density there was increasing risk of 0.61 (SE = 0.20) (p = 0.0018). In a model comparing to the Infection Positive profile, Pre-Positive individuals had an estimated risk of -1.397 (SE = 0.62; p = 0.0239), after controlling for the increased risk of febrile illness with increasing parasite density (GEE, p = 0.0039).

## Discussion

In a low and recent falciparum malaria transmission area of the Peruvian Amazon, the *P. falciparum *infection prevalence as well as the αMSP1-19kD IgG seroprevalence were measured during community surveys. In addition, the dynamics of IgG responses before, during, and after *P. falciparum *infections and how this is related to asymptomatic infections were evaluated in both children and adults.

Similar to historic data [12,26] reported since 2000, parasite prevalence was found to be low during the 2003 and 2004 transmission seasons. When using active as well as passive case detection the maximum *P. falciparum *infection rate was only 0.42 infections/person/year[12]. However, a limitation of this study is that ACD was not conducted during the four month dry season. The reason for this was that passive case detection was done throughout the year and that all previously collected historic clinical and entomologic data showed negligible malaria transmission during the dry season[12]. As mentioned earlier, to remedy this issue, a small community survey of 238 individuals was conducted during the dry season of 2007. Not a single *P. falciparum *infection was found (as determined by both microscopy and PCR).

Despite this low transmission, the αMSP1-19kD IgG seroprevalence was comparable in the beginning versus the end of malaria transmission season in both children (27%–30%) and adults (53%–65%). Furthermore, the median αMSP1-19kD IgG level was very similar in *Plasmodium*-negative and *P. falciparum*-positive adults. This is likely attributable to infections suffered in months or years in the past, suggesting that αMSP1-19kD IgG responses are maintained in the majority of individuals.

It is clear that the αMSP1-19kD response in both children and adults in this cohort can last longer than the 1–4 months consistently described in high transmission studies[3,6,7,9,10,22]. It is important to note that the presence of *P. vivax *transmission in this area [12] could not explain these results. As shown in this study, and in other results from this laboratory, the αMSP1-19kD IgG responses were *P. falciparum*-specific.

Finding children at the end of the 4-month low transmission season with antibodies shows that some children had antibody lasting more than 4 months. However, *P. falciparum*-negative children had a median αMSP1-19kD IgG of 0.301, which was clearly lower than *P. falciparum*-positive children (1.34) or *P. falciparum*-positive adults (1.45). This is consistent with an age-related increase in cumulative life time prior *P. falciparum *infections.

The accumulation of *P. falciparum *infections with age in Peru is unlike that in high transmission regions, where all individuals likely have had many infections during their first years of life. In high transmission regions the observed antibody responses do not reflect classical response dynamics described in immunology textbooks[27]. Instead, they are short-lived as though the primary response is reinitiated, and the antibody secreting cells seem to quickly undergo cell death after each infection[3,7,10,28,29]. In contrast, it is expected that if immunologic memory develops with exposure/age, older individuals would have an IgG response before infection and a more rapid increase in IgG level upon infection. This is exactly what was detected when comparing the Before, During, and After αMSP1-19kD responses with age in our Peruvian cohort. Because sampling date was not dependent upon individuals coming to the clinic, the results indicate that children 0–6 years-old were slower to increase antibody levels in comparison to adults. Again, this is consistent with the probability of prior *P. falciparum *infection(s).

However, the age-related differences in seroprevalence, IgG level when one is *P. falciparum *negative, and response dynamics upon infection are also consistent with children's intrinsically lower capacity for eliciting and maintaining antibody responses that continues until approximately 12 years of age[30,31]. These less well-maintained responses of children were suggested by their seroprevalence being lower than that of adults despite similar *P. falciparum *infection prevalences during this two year study. In fact, of the seven individuals who were repeatedly sampled in beginning (February 2004) and ending (August 2004) malaria season community surveys, and who were IgG Negative in February 2004 but became infected during the 2004 season, the only two individuals who were not IgG positive in the August 2004 community survey were children.

Similarly, as concluded by Tongren et al. in 2006[32] and Akpogheneta et al. in 2008[33], our studies have found that age is associated with a maintained seroprevalence. In this study's Peruvian cohort, during 2003 and 2004, the parasite prevalence was approximately 1% [12]. In the 4 month dry season of The Gambia, Africa, αMSP1-19kD IgG responses lasted less than 4 months in all age groups tested and less than 2 months in children less than 5 years old. In this Peruvian cohort, priorly infected children less than 5 years old had antibody responses lasting greater than 4 months while the response in priorly infected adults appeared even more long-lived.

Although our IgG subclass analysis has not yet been completed, it is interesting to consider that if IgG subclass switching predicts longer duration antibody production, then the results of Tongren et al. suggest that both age and transmission intensity at some threshold between 1% and 18% are associated with more IgG1 and theoretically longer lived total αMSP1-19kD IgG antibody responses[32]. Akpogheneta et al. reported subclass responses to malaria antigens, although antigens other than αMSP1-19kD, and found that older individuals (individuals likely having had more prior exposure history) had a more maintained IgG1 response[33]. Supporting this suggestion, a study of 0–2 year-olds in Kenya found that children who received *P. falciparum *infections later in life had higher levels of αMSP1-19kD IgG and an IgG subclass ratio more associated with protection (mostly IgG1 and IgG3; not IgG2 and IgG4)[34].

Furthermore, in our study clinical symptoms of malaria were associated with a pre-infection antibody response (Table 3). A Pre-Positive αMSP1-19kD IgG response was associated with lower frequency of fever as well as a lower parasite density, in both adults and children. This emphasis on the Before infection antibody response is similar to prior studies demonstrating that the antibody response one month before an infection is often the best predictor of a sub-clinical, low density infection[7,33].

Whether related to age at first infection and/or the slow accumulation of prior *P. falciparum *infections, it is clear that the αMSP1-19kD response in both children and adults in this cohort can last longer than the 1–4 months consistently described in high transmission studies[3,6,7,9,10,22]. It is possible that this immune capacity is specific to this population of Peruvians (host-specific factors) or *P. falciparum *parasites (parasite-specific factors). Another possibility is that spaced infections in low transmission areas prime the immune response in a manner that is especially effective for developing anti-malaria immunity. This more generalized hypothesis is supported by the few studies of antibody responses comparing populations in low transmission regions versus high transmission regions. A study from Brazil showed that individuals living in areas of lower transmission had higher anti-*P. falciparum *immune responses than a neighboring village with higher *P. falciparum *transmission[21]. Similarly, Mlambo *et al *were surprised to find higher IgG levels in populations living in low transmission regions versus those in higher transmission for 3 (including MSP1-19kD) of 5 vaccine candidate antigens tested[22].

In conclusion, despite the short-lived responses observed in high transmission studies, at least in some populations (such as our low transmission Peruvian cohort) it is possible to naturally acquire a maintained, clinically relevant antibody response against *P. falciparum*, which is an encouraging sign for all malaria vaccine initiatives.

## List of abbreviations used

MSP1-19kD: Merozoite Surface Protein-1 19kD; OD: Optical Density; ACD: Active Case Detection; PCD: Passive Case Detection; PCV: Packed red Cell Volume; PCR: Polymerase Chain Reaction.

## Competing interests

The authors declare that they have no competing interests.

## Authors' contributions

KJT carried out the majority of experiments, participated in the statistical analysis, and began the manuscript. EC analyzed data from the immunoassays as well as the clinical data, performed statistical analyses, and drafted the manuscript. JH participated in construction and coordination of the study protocol and lead sample collection. KS and DG assisted in sample preparation and organization, over sight in experiments and reviewed the manuscript. OHB conceived of the study, performed statistical analyses, participated in the study design and coordination, and helped to draft the manuscript. All authors read and approved the final manuscript.
